# Change of urban and rural construction land and driving factors of arable land occupation

**DOI:** 10.1371/journal.pone.0286248

**Published:** 2023-05-31

**Authors:** Suxia Zhao, Mengmeng Yin

**Affiliations:** School of Surveying and Land Information Engineering, Henan Polytechnic University, Jiaozuo, Henan, China; PLOS ONE, UNITED KINGDOM

## Abstract

Under the background of global urbanization, the continuous expansion and extensive utilization of urban and rural construction land has caused a large amount of arable land to be occupied, which seriously threatens national food security. This paper describes the spatio-temporal patterns of urban and rural construction land expansion and its occupation of arable land by using the urban and rural construction land expansion intensity, the urban and rural construction land expansion intensity difference index, and geo-detector model. It also explores the mechanisms through which the arable land was occupied. Results showed that construction land in both urban and rural areas expanded over the period 2009–2018 despite a large number of rural and urban migrants, and the major contributor to the rapid urbanization in China. This dual expansion could mainly be attributed to the tendency of these migrants to keep or even enlarge their rural construction land, which also resulted in a severer arable land loss than that caused by the expansion of urban construction land. Second, the rate of arable land occupied by urban and rural construction land in Henan province has been gradually slowing down, whereas the expansion of rural construction land is most dependent on arable land occupation. Third, according to the geo-detector model, the relationship between urbanization level and arable land occupied by urban and rural construction was the strongest, followed by the growth rate of fixed asset investment and the proportion of secondary and tertiary industries in GDP.

## 1. Introduction

The agglomeration and expansion of urban space tend to evolve dynamically in terms of economic and population spatial distribution [1], and both are primarily reflected in the change of urban and rural construction land in material space. Urban and rural construction land is the main carrier of human non-agricultural economic activities, and its evolution is the inevitable trend of economic development [2]. At this stage, the urbanization process of small and medium-sized cities will continue to advance, the urban economy will further develop, and urban and rural construction land will continue to expand. However, with the rapid development of urbanization, a series of problems related to urban and rural land use have also emerged. First, as the urbanization rate of the population continues to increase, the rural population will enter the city in greater numbers, the urban scale will continue to inflate, and the construction land will expand rapidly, resulting in the conversion of a large amount of arable land around the city into construction land, highlighting the contradiction between arable land protection and urbanization development. Second, due to the large number of rural people flowing into cities, land used for rural construction has been left idle and is being wasted. At the same time, the existence of idle, desolate, and unplanted arable land has further intensified the contradiction of arable land. Third, with the introduction of a series of policy dividends, such as having a beautiful countryside and rural revitalization, there has been a wave of housing construction in rural areas, which has led to an increase in rural construction land, and a synergistic growth in both urban and rural construction land is now occurring. The problems of rural population reduction and an increase in land [3].

The coronavirus disease 2019 pandemic has aroused concerns in the community regarding national food security. The stable quantity of arable land resources is a fundamental factor in ensuring national food security [4, 5]. China’s rapid economic growth and the process of urbanization have attracted the attention of the world [6, 7]. The acceleration of China’s urbanization process has further strengthened its dependence on urban and rural construction land. In terms of economic theory, the improvement of urban land value stems from the attraction and agglomeration of rural populations by urbanization, which leads to the reduction of rural construction land and the intensive development of urban land [8]. However, under the background of urbanization with a large population, the land designated as rural construction land is increasing instead of decreasing. Although the implementation of the policy of linking the increase and decrease in urban construction land has been alleviated to a certain extent, the internal structural contradiction caused by the simultaneous increase in both urban and rural construction land remains severe. The current situation of land use runs counter to the process of urbanization. Urban and rural construction land leech from arable land on both sides, and most of the land being lost is high-quality arable land, which seriously threatens national food security. Therefore, revealing the expansion characteristics of urban and rural construction land and the driving mechanism of occupied arable land is key to the sound development of arable land protection and urbanization in China.

In recent years, experts and scholars have conducted a lot of research on the expansion of urban and rural construction land and the loss of arable land and have gathered fruitful results. Available research on urban and rural construction land has mainly focused on such aspects as the expansion of urban construction land [9–17], the spatio-temporal evolution of rural construction land [18–20], and the linkage between the increase and decrease in urban and rural construction land [21–23]. The driving force analysis is mainly reflected in the impact of natural, social, economic, population, policy, and landscape patterns on urban and rural construction land [24–32]. Meanwhile, research on arable land occupation to date has mainly focused on the temporal and spatial characteristics and driving factors of arable land loss [33–36], the relationship between the loss of arable land and urban expansion and economic development [37–40], and the formation mechanism of urban and rural construction land occupation of arable land [6, 41]. However, few simultaneous studies on the expansion of urban and rural construction land and the occupation of arable land and few in-depth analyses from the perspective of geographic space exist even now.

In view of this, the present paper focuses on urban and rural construction land as the main bearing space of population and economic activities, selecting Henan province with its rapid urbanization and obvious regional economic gradient differences as the study area. This paper first explores the inherent relationship between urban and rural construction land expansion and arable land protection, then tries to reveal the occupation characteristics and driving mechanism of various land types within urban and rural construction land and provide a scientific basis for the rational allocation of regional urban and rural construction land and the promotion of the sustainable use of arable land.

## 2. Materials and methods

### 2.1 Study area

**Fig 1:**
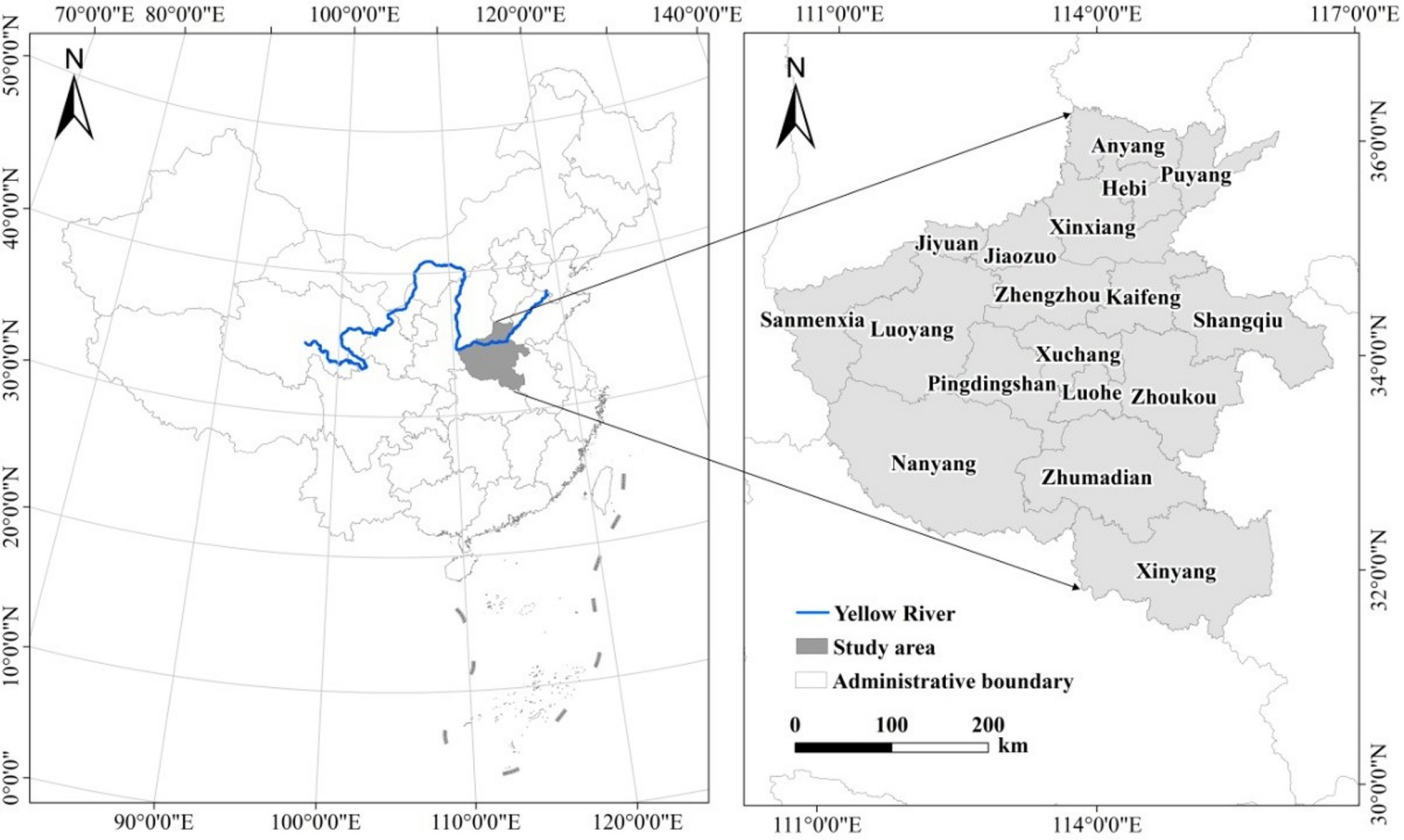
Location of the study area. Note: All administrative boundary data comes from Resource and Environment Science and Data Center (https://www.resdc.cn/).

Henan province is located in the middle and lower reaches of the Yellow River, with a total area of 167,000 km^2^, accounting for 1.73% of the total area of the country (Fig 1). By the end of 2018, Henan province had a registered population of 109.06 million and a permanent population of 96.05 million, including 56.39 million urban residents and 52.67 million rural residents. The population urbanization rate was 51.71%, which is far lower than the national rate of 59.58% measured during the same time period. The amount per capita of construction land in urban areas is 116.92m^2^, while the amount per capita of construction land in rural areas is 302.43m^2^, and the problem of extensive utilization of urban and rural construction land is prominent. Henan province is selected as the case area to study the relationship between urban and rural construction land and arable land occupied, with the following characteristics: (1) Henan province is a major agricultural province and a food security base in China. It bears the major responsibility to ensure the national food production safety. The conflict of land resource occupation caused by farmland protection and urbanization is serious. (2) The urbanization of Henan province has a low starting point and great potential. Since the urbanization rate exceeded 30% in 2005, it has entered a period of rapid urbanization and accelerated urban and rural transformation. The relationship between urban and rural population and arable land change is in a stage of drastic change.

### 2.2 Data sources

Considering the consistency of the data, 2009∼2018 was selected as the research period. The data of urban and rural construction land were derived from the data of the second national land survey and the annual change data of land use (https://www.resdc.cn/DOI/DOI.aspx?DOIID=54). The gross domestic product (hereinafter called GDP) change rate, the per capita GDP change rate, the growth rate of secondary and tertiary industries, the change rate of total fixed asset investment of the whole society, the increment of urbanization rate, and the population change rate were taken from the statistical yearbook of Henan province (2010∼2019) (https://www.henan.gov.cn/zwgk/zfxxgk/fdzdgknr/tjxx/tjnj/).

### 2.3 Research methods

Based on previous studies [8, 42–46], this paper mainly adopted the research methods of urban and rural construction land expansion intensity, the urban and rural construction land expansion intensity difference index, and geo-detector. Among them, urban and rural construction land expansion intensity and the urban and rural construction land expansion intensity difference index were used as measures to analyze the spatio-temporal change characteristics of urban and rural construction land in Henan province. The geo-detector was used to diagnose the obstacle factors affecting the changes of urban and rural construction land from the perspective of geographical spatial distribution.

#### 2.3.1 The urban and rural construction land expansion intensity index

The urban and rural construction land expansion intensity index used the urban land expansion intensity index model to reflect the expansion characteristics of urban and rural construction land in different time periods [42].


$$UEI_{n}=\;\frac{{\text{A}}_{\text{n}}^{{\text{t}}_{2}}-{\text{A}}_{\text{n}}^{{\text{t}}_{1}}}{{\text{A}}_{\text{n}}^{{\text{t}}_{1}}\times \Delta \text{t}}\cdot$$ (1)

Where *UEI*_*n*_ represents the urban and rural construction land expansion intensity index; ${\text{A}}_{\text{n}}^{{\text{t}}_{1}}$ and ${\text{A}}_{\text{n}}^{{\text{t}}_{2}}$ represent the urban and rural construction land area of a city in *t*_*1*_ and *t*_*2*_, respectively; and Δt represents the interval year from *t*_*1*_ to *t*_*2*_.

#### 2.3.2 Urban and rural construction land expansion intensity difference index

The urban and rural construction land expansion intensity difference index is the ratio of the change intensity of urban and rural construction land expansion in a city to that in all cities, reflecting the difference of urban and rural construction land expansion intensity in different cities [42]. The index can be used to perform a horizontal comparison of the expansion of urban and rural construction land in various cities, to exclude the influence of the size of each city, and to compare the difference in the expansion intensity of urban and rural construction land in different cities in the same period.


$${\text{UEDI}}_{\text{n}}=\frac{\left|{\text{A}}_{\text{n}}^{{\text{t}}_{2}}-{\text{A}}_{\text{n}}^{{\text{t}}_{1}}\right|\times {\text{A}}^{{\text{t}}_{\text{1}}}}{\left|{\text{A}}^{{\text{t}}_{\text{2}}}{\text{-A}}^{{\text{t}}_{\text{1}}}\right|\times {\text{A}}_{\text{n}}^{{\text{t}}_{\text{1}}}}$$ (2)

Where *UEDI*_*n*_ represents the urban and rural construction land expansion intensity difference index and ${\text{A}}^{{\text{t}}_{2}}$ and ${\text{A}}^{{\text{t}}_{1}}$ represent the total urban and rural construction land area of Henan province in *t*_*1*_ and *t*_*2*_ time periods, respectively.

#### 2.3.3 Geo-detector

Geo-detector is a group of statistical methods that use spatial data to detect the spatial differentiation of geographical elements or geographical phenomena to reveal the driving factors behind them [44–46]. This paper intends to introduce a model to detect the main driving factors of urban and rural construction land occupation of arable land, where the magnitude of the factor driving force is measured using the q value. The calculation formula is as follows:

$$q=1-\frac{1}{N\sigma^{2}}\sum_{h=1}^{L}N_{h}\sigma_{h}^{2}$$ (3)

Where *q* is the degree of influence of the detection factor on the change of urban and rural construction land occupied by arable land; *N*_*h*_ and $\sigma_{h}^{2}$ are the number of units and variance in the secondary study area; and *N* and *σ* are the number of units and variance of the whole study area, respectively. The value range of *q* is *0–1*; when *q = 0*, it indicates that the detection factor has no influence on the change of arable land occupied by urban and rural construction land, while, when *q = 1*, it indicates that the detection factor has absolute control over the change of arable land occupied by urban and rural construction land. The larger the value of *q*, the stronger the driving effect of the detection factor on the change of arable land occupied by urban and rural construction land.

## 3. Results and discussion

### 3.1 The evolution characteristics of the spatiotemporal pattern of urban and rural construction land

#### 3.1.1 Changes in urban and rural construction land

**Fig 2:**
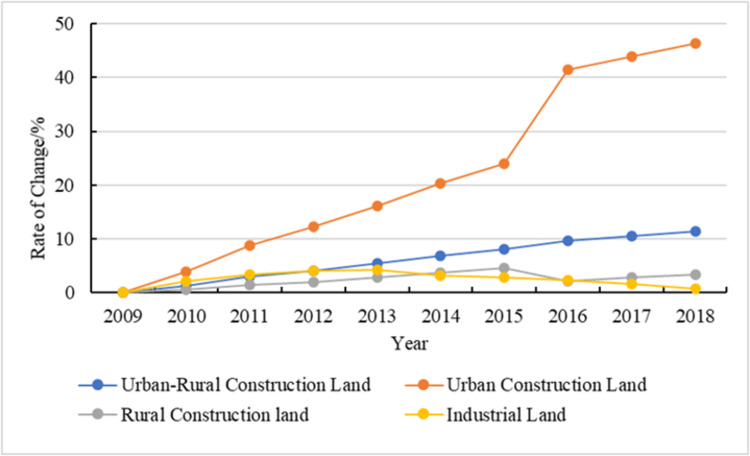
Trend chart of urban and rural construction land change rates in Henan province from 2009–2018.

The urban and rural construction land in Henan province increased by 2306.2 km^2^ from 2009–2018 and showed an upward trend (Fig 2). Among the types of land, the scale of urban construction land increased the fastest; the area of new urban construction land accounted for 76.62% of the total growth of urban and rural construction land and increased sharply in 2016, while the land selected for rural construction land decreased, mainly due to the implementation of the policy of linking urban and rural construction land, and some rural construction land was also converted to urban construction land. Across the whole research period, the increase in urban construction land area was accompanied by the process of population urbanization and land urbanization in Henan province. In recent years, the average annual urbanization level in Henan province has increased by >1.5 percentage points, helping to promote a continuous increase in urban construction land.

Second, rural construction land is also expanding, but the growth rate has slowed down since 2016. The cumulative increased area accounted for 23.06% of the total growth of urban and rural construction land from 2009–2018. With the continuous improvement of the urbanization process, a large number of rural people are migrating to cities, but a large number of farmers who occupy land in both urban and rural areas still exist today.

#### 3.1.2 Expansion intensity of urban and rural construction land

The expansion intensity of urban and rural construction land in Henan province generally showed a downward trend, exhibiting phased characteristics (Table 1). The maximum expansion intensity of urban and rural construction land was 1.37 from 2009–2012, then decreased to 1.01 from 2015–2018. From the perspective of various types of land use, the expansion intensity of urban construction land was the largest and showed an upward trend. The land use of rural construction land showed an expansion trend from 2009–2015, but during 2015–2018, the land use of rural construction land experienced a decreasing trend.

**Table 1:** Index table of expansion intensity of urban-rural construction land in Henan province from 2009–2018.

Year	Urban and rural construction land	Urban construction land	Rural construction land
2009–2012	1.37	4.08	0.69
2012–2015	1.30	3.49	0.81
2015–2018	1.01	5.99	−0.35
2009–2018	1.27	5.14	0.38

**Fig 3:**
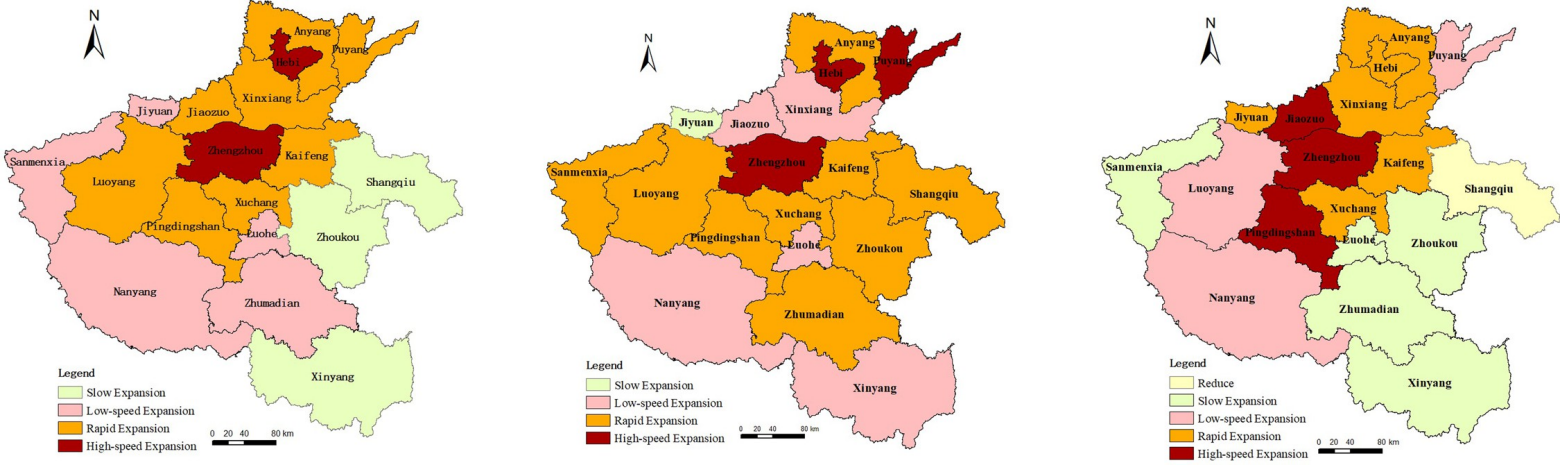
The intensity of distribution of urban and rural construction land expansion in Henan province from 2009–2018. Note: All administrative boundary data comes from Resource and Environment Science and Data Center (https://www.resdc.cn/).

In terms of space, there are significant spatial variations in urban and rural construction land due to the great differences in natural, economic, and population factors. We calculated the difference index of the urban and rural construction land expansion intensity of each city based on the city scale, then used the natural break point classification method to divide the expansion intensity of each type of land into the following five major types: slow expansion, low-speed expansion, medium-speed expansion, rapid expansion, and high-speed expansion [28]. As can be seen from Fig 3, there are differences in the expansion of urban and rural construction land among cities, showing a trend of unbalanced layout on the whole. The areas with rapid expansion of urban and rural construction land are mainly located in Central and Northern Henan, with rapid expansion in Zhengzhou and Hebi. In contrast, Xinyang district, Zhoukou district, and Shangqiu district expanded more slowly.

There were also differences in the expansion degrees of urban construction land and rural construction land. Considering urban construction land, Zhengzhou district, Hebi district, and Puyang district expanded rapidly. As the capital city of Henan province, Zhengzhou district increased its urban construction land content the fastest, followed by Luoyang district, Xinxiang district, Nanyang district, Zhoukou district, and Zhumadian district, while Jiyuan district increased its urban construction land content the sloweast. It can be seen that the expansion of urban construction land is highly related to the spatial pattern of economic development and population agglomeration. Except for Shangqiu district, the land used for rural construction land is showing an expansion trend. The high-speed expansion areas are mainly located in Pingdingshan district, Zhengzhou district, and Jiaozuo district. As can be seen from Fig 3, the internal structural contradiction of simultaneous growth of urban construction land and rural construction land is still severe.

### 3.2 Driving mechanism of arable land occupation for urban and rural construction land in Henan province

#### 3.2.1 Change characteristics of arable land occupied by urban and rural construction land

According to statistics (Table 2), urban and rural construction land in Henan province occupied 2038.5km^2^ of arable land from 2009–2018, accounting for 76.47% of the total area of new urban and rural construction land. Among the 2038.5km^2^ arable land occupied from 2009–2018, the proportion of arable land occupied by urban construction land was 49.64%, that occupied by rural construction land was 57.03%. Among the two types of urban and rural construction land, the rural construction land occupied 1026.5km^2^ of arable land, which is much larger than its expansion area (531.8km^2^), which was 1.93 times the expansion area of rural construction land. This feature shows that, in the context of rapid urbanization, urban construction land has occupied surrounding rural construction land in the process of outward expansion, and the expansion of rural construction land has occupied more arable land. Therefore, although the total expansion of rural construction land is limited, the occupation of arable land is very serious.

**Table 2:** Table of arable land occupied by urban and rural construction land in Henan province from 2009–2018.

Land use type	Net increment of urban and rural construction land(km^2^)	Occupation of arable land		Ratio of arable land occupied to net increment(%)
		Area(km^2^)	Structure(%)	
Urban construction land	1774.40	1012	49.64	57.03
Rural construction land	531.80	1026.50	50.36	193.05
Urban and rural construction land	2306.20	2038.50	100.00	88.39

#### 3.2.2 Driving mechanism of arable land occupied by urban and rural construction land

**Fig 4:**
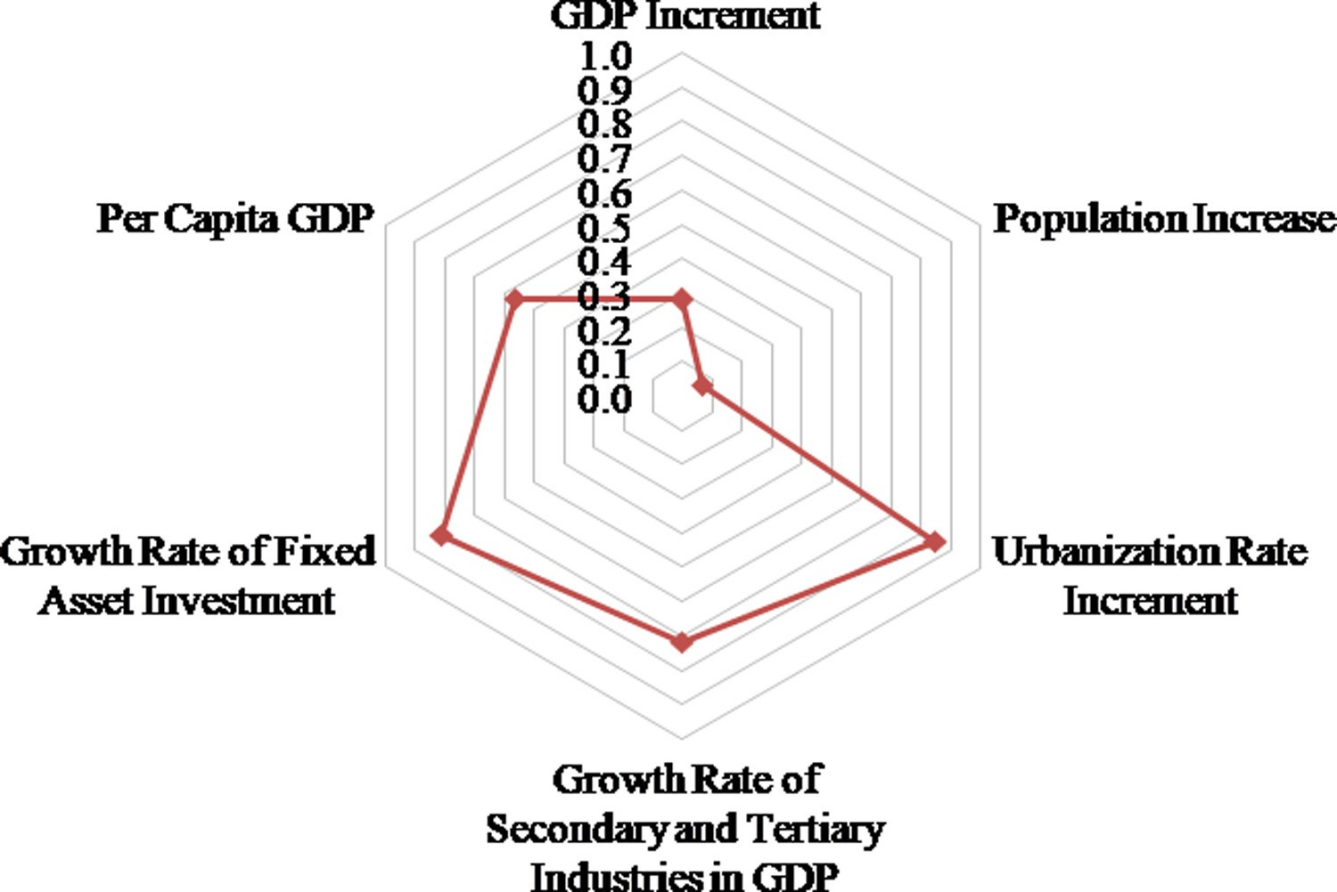
A radar map of the main driving factors of arable land occupied by urban and rural construction land in Henan province.

Urban and rural construction land is where most of human social and economic activities take place [10]. The change of urban and rural construction land is affected by many factors, such as natural conditions, economic development level, social living conditions, social investment environment, and policy factors. Among them, natural factors and policy factors are not easy to quantify, but these factors can jointly drive the expansion of urban and rural construction land by influencing the level of social and economic development. Therefore, the paper uses existing research results [35, 39], combined with the actual situation of Henan province, to select the GDP change rate, the per capita GDP change rate, the growth rate of secondary and tertiary industries, the change rate of total fixed asset investment of the whole society, the increment of urbanization rate, and the population change rate in Henan province from 2009–2018 as the detection impact factors of arable land occupied by urban and rural construction land (Fig 4).

The geo-detector model was used to calculate the impact degree of the impact factors of urban and rural construction land occupation of arable land. It can be seen from Fig 4 that the urbanization level has the greatest impact on the urban and rural construction occupation of arable land, with an impact index of 0.8495, indicating that, with the continuous improvement of the level of urbanization. The continuous expansion of urban construction land has taken up a large amount of arable land. After further investigation, it was found that, in areas where urban construction land is expanding rapidly, land designated for rural construction land has also grown rapidly, resulting in a further increase in the amount of arable land occupied. The growth rate of fixed asset investment, the growth rate of the secondary and tertiary industries in GDP, and the per capita GDP also have had a strong influence on the amount of arable land occupied by urban and rural construction, and the driving effect of fixed asset investment on the arable land occupied by urban and rural construction is stronger than the effect of GDP growth. This shows that the current economic structure of Henan province is not reasonable. It mainly relies on investment to drive economic growth. The pressure of resource consumption is too large, and the pressure and difficulty of protecting arable land will also increase. In addition, the arable land occupied by urban and rural construction has a certain relationship with population growth, but the correlation is relatively small.

#### 3.2.3 Differentiation mechanism of dominant factors of arable land occupied by urban and rural construction land

On the basis of quantitatively detecting the dominant factors of the spatial differentiation of arable land occupied by urban and rural construction in Henan province, this paper further explores the dominant factors of urban and rural construction in Henan province using the following three dominant factors: urbanization rate, fixed asset investment, and the proportion of secondary and tertiary industries in GDP. The internal mechanism of the factor will help the rational allocation of urban and rural construction land and the protection of arable land.

**Fig 5:**
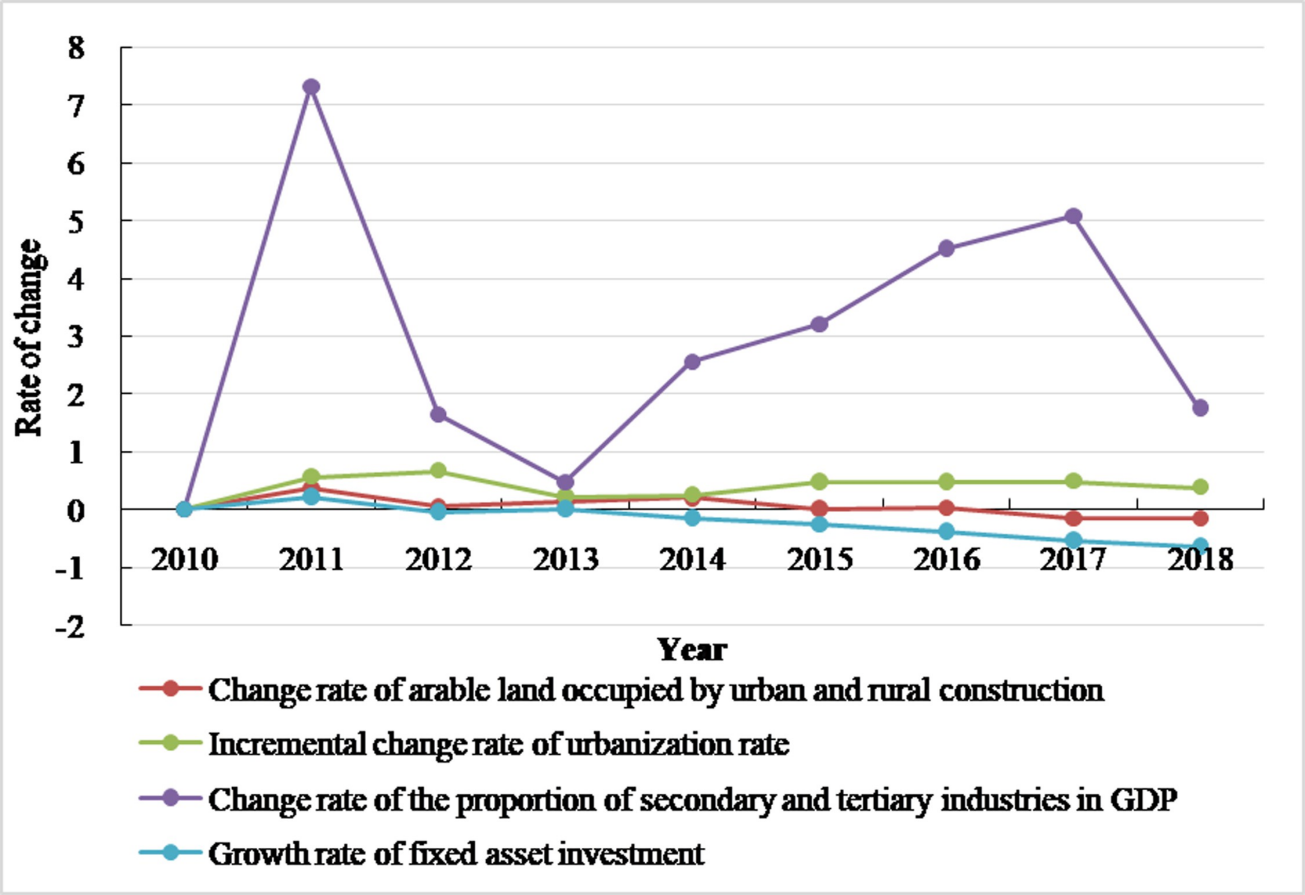
Trend map of arable land occupation and driving forces in urban and rural construction land in Henan province from 2009–2018.

First, the urbanization rate rose from 37.7% to 51.71% from 2009–2018, representing an increase of 14.01%, with an average annual increase of 1.56%. It can be seen from Fig 5 that, since 2009, the urbanization rate has gradually slowed down, and the rate of increase of arable land occupied by urban and rural construction has also gradually slowed down, while the change trend of the urbanization rate has remained basically consistent with the rate of arable land becoming occupied by urban and rural construction. This shows that the land-saving effect of urbanization in Henan province has not been brought into play. The land designated for rural construction land has not decreased with emigration of the rural population to the city, but the area has expanded. Urban and rural construction land dually occupy arable land, which intensifies the phenomenon of arable land occupation in the process of urbanization.

Second, in 2018, investment in fixed assets in Henan province reached 470.1663 billion yuan, representing an increase of 33327.27 billion yuan over 2009, with an average annual increase of 370.303 billion yuan, or 2.43 times that of 2009. It can be seen from Fig 5 that the change rate of fixed asset investment in Henan province is generally consistent with the change rate of urban and rural construction in arable land from 2009–2018, indicating that, in the process of urbanization, with the increase in fixed asset investment, relevant infrastructure has been continuously improved, construction land has been continuously increased, a large amount of arable land has been occupied to a certain extent, and the amount of arable land has decreased. In addition, with the improvement of people’s living standards, people’s demand for land for infrastructure, such as tourism, leisure, and entertainment, is also increasing, and part of the arable land has been occupied in response.

Finally, with the continuous acceleration of urbanization, the increase in fixed asset investment of the whole society has benefitted the secondary and tertiary industries. In 2009, the proportion of secondary and tertiary industries in Henan’s GDP increased from 86.36% to 91.07% in 2018. It is not difficult to see that the rate of change in the proportion of the secondary and tertiary industries in GDP has an obvious positive correlation with the repurposing of arable land for urban and rural construction. The main reason for this is that the Henan provincial government has attached great importance to the development of secondary and tertiary industries in recent years, which has led to the occupation of a large amount of arable land.

## 4. Discussion

According to the general understanding, in the urbanization stage, with the continuous emigration of rural populations to cities, the demand for urban land will continue to increase, and the scale of urban construction land will inevitably expand. At the same time, with the continuous movement of farmers into cities, the rural population is greatly reduced, the demand for rural housing and infrastructure is less, and the scale of rural construction land should be continuously reduced. The process of urbanization is not only the process of increasing urban construction land and decreasing rural construction land but also the process of reducing the total amount of urban and rural construction land and increasing the degree of intensive land use and saving. This is the general mechanism of construction land growth in the process of urbanization.

Surprisingly, this "general mechanism" does not seem to play a role in the process of urbanization in China. Through studying the change trend of urban and rural construction land in Henan province from 2009–2018, we found that, in the process of rapid migration of a rural population to urban and rural areas, the amount of rural construction land "increased without reduction," and both urban and rural construction land together showed a phenomenon of "synergistic growth". The reasons for this are as follows: first, while the rural resident population is decreasing, a large number of rural homesteads have not ceased to exist, but a large amount of village land is being abandoned or left idle, resulting in the hollowing out of villages; Second, the original construction land in rural areas cannot be effectively decommissioned, and the amount of newly added construction land is continuously increasing. Therefore, a phenomenon of "the numbers of people are decreasing and the amount of land is increasing" in rural areas has been created. The land-saving effect of urbanization cannot be brought into play. A large amount of arable land is occupied by rural construction land and is showing a rising trend. This is the main source of the continuous expansion of China’s urban and rural construction land and the occupation of a large amount of arable land.

Compared to similar studies, this paper can combine the expansion of urban and rural construction land with the loss of arable land, and the research perspective is richer. The research results have important theoretical significance and practical value for the rational allocation of urban and rural construction land and in efforts to ensure China’s food security. This study provides effective support for scientifically delimiting urban development boundaries in land spatial planning, preventing disorderly urban spread, improving land use efficiency, and promoting the transformation of urban development from extension expansion to connotation development. At the same time, it also contributes to world food security while simultaneously ensuring China’s national food security.

There are still some deficiencies in this paper, which need to be further studied and discussed. First, in terms of the analysis of driving factors of arable land occupied by urban and rural construction land, the evaluation indicators constructed in this paper only considered six representative evaluation indicators, such as the increment of urbanization rate and the rate of population change, due to limitations of the data. In fact, the behaviors of the government, enterprises, and residents also have an important impact on the arable land occupied by urban and rural construction. These factors should be considered in the future to improve the scientificity of the evaluation results. Second, in terms of the expansion of urban and rural construction land, this paper mainly considers the measurement of urban and rural construction land structure but does not consider its layout measurement, and it fails to more accurately grasp the spatial differences of urban and rural land use change. In the future, the two factors of urban and rural construction land structure and layout measurement should also be comprehensively considered, which will help us to obtain a deeper and comprehensive understanding of the internal mechanism of urban and rural construction land change in China.

## 5. Conclusion

Since 2009, the expansion intensity of urban and rural construction land in Henan province has generally shown a downward trend. From the perspective of various land types, the expansion intensity of urban construction land is the largest and shows an upward trend. From the perspective of cities in Henan province, the cities are where urban construction land has increased, and the amount of land designated as rural construction land is also increasing. The phenomenon of "synergistic growth" of urban and rural construction land is widespread, and the degree of intensive utilization of urban and rural construction land is relatively low.

In terms of the loss of arable land occupied by urban and rural construction land, the occupation of arable land by rural land is the most serious. The rural construction land occupies 1026.5km^2^ of arable land, which is much larger than its expansion area (531.8km^2^), and this is 1.93 times the expansion area of rural construction land. This feature shows that, in the context of rapid urbanization, urban construction land has occupied surrounding rural construction land in the process of outward expansion, and the expansion of rural construction land has occupied more arable land. Therefore, although the total expansion of rural construction land is limited, the occupation of arable land is very serious.

Additionally, it was found that the level of urbanization has the greatest correlation with the arable land occupied by urban and rural construction through the geo-detector model. It was shown that the urbanization model is very important for urban and rural construction and the temporal, spatial, and structural characteristics of the arable land occupied. Second, fixed asset investment and the development of secondary and tertiary industries are the main driving forces for the growth of urban and rural construction land, which dominate the temporal and spatial patterns of urban and rural construction and the occupation of arable land. Finally, there is a certain correlation between population growth and the proportion of arable land occupied by urban and rural construction, but the correlation is small.
